# Co-thought gesturing supports more complex problem solving in subjects with lower visual working-memory capacity

**DOI:** 10.1007/s00426-018-1065-9

**Published:** 2018-07-31

**Authors:** Charly Eielts, Wim Pouw, Kim Ouwehand, Tamara van Gog, Rolf A. Zwaan, Fred Paas

**Affiliations:** 1 Department of Psychology, Education, and Child Studies, Erasmus University Rotterdam, 3000 DR Rotterdam, The Netherlands; 2 Department of Psychological Sciences, University of Connecticut, Storrs, USA; 3 Department of Pedagogical and Educational Sciences, Utrecht University, Utrecht, The Netherlands; 4 School of Education/Early Start, University of Wollongong, Wollongong, Australia

## Abstract

During silent problem solving, hand gestures arise that have no communicative intent. The role of such co-thought gestures in cognition has been understudied in cognitive research as compared to co-speech gestures. We investigated whether gesticulation during silent problem solving supported subsequent performance in a Tower of Hanoi problem-solving task, in relation to visual working-memory capacity and task complexity. Seventy-six participants were assigned to either an instructed gesture condition or a condition that allowed them to gesture, but without explicit instructions to do so. This resulted in three gesture groups: (1) non-gesturing; (2) spontaneous gesturing; (3) instructed gesturing. In line with the embedded/extended cognition perspective on gesture, gesturing benefited complex problem-solving performance for participants with a lower visual working-memory capacity, but not for participants with a lower spatial working-memory capacity.

## Introduction

Most research on the cognitive function of gestures has focused on co-speech gestures: hand gestures that are synchronized with and meaningfully related to speech (e.g., Cook, Yip, & Goldin-Meadow, 2012; Goldin-Meadow, Nusbaum, Kelly, & Wagner, 2001; Marstaller & Burianová, 2013). This research has revealed that participants who gestured while explaining a solution of a math or physics problem performed better on a concurrent secondary task (e.g., remembering strings of letters or 2-d spatial coordinates) compared to participants who did not gesture, suggesting that gesturing participants have more cognitive resources available than participants who are inhibited to gesture.

If gestures do, indeed, provide cognitive resources, what do these resources consist of? With regard to co-speech gestures, there is the hypothesis that these gestures support semantic processes (e.g., Cook et al., 2012). For example, during verbal explanation of a problem solution, some of the information that needs to be communicated is more efficiently conveyed by depicting it through gesture than in an analog format through speech. As such, distributing communicative content over multiple modalities would reduce the effort needed for speech production (Cook et al., 2012).

In the present study, we investigated how non-communicative gestures produced without speech (i.e., co-thought gestures) may support cognitive resources. Hand gesticulations, such as pointing to objects or acting on imagined objects, not only arise in communicative contexts, but also in a wide variety of problem-solving contexts. For example, Chu and Kita (2008, 2011, 2016) have shown repeatedly that participants who are allowed and/or encouraged to gesture, perform better on a mental rotation task than participants who were not allowed to gesture. In their paradigm, participants judged whether two objects are the same when presented under different orientations. Chu and Kita (2008, 2011, 2016) found that problem solvers produced gestures as-if actually rotating the object to match the target object. Participants produced these gestures when they verbally explained their problem solution, but also during silent problem solving, even when silent speech processes were inhibited by a secondary task (Chu & Kita, 2008, 2011, 2016).

Furthermore, co-thought gestures are also consistently found to boost performance on other tasks, such as counting coins (Kirsh, 1995), mental abacus calculations (Brooks, 2014), tracking moving items in space (Delgado, Gómez, & Sarriá, 2011; Logan, Lowrie, & Diezmann, 2014; Macken & Ginns, 2014; So, Ching, Lim, Cheng, & Ip, 2014), solving fraction problems (Zurina & Williams, 2011), route learning (e.g., Logan et al., 2014; So et al., 2014), and rotating gear problems (e.g., Alibali, Spencer, Knox, & Kita, 2011; Stephen, Dixon, & Isenhower, 2009). The fact that these gestures also spontaneously occur without communicative intent suggests that, at least in some cases, gestures have a cognitive function that goes beyond supporting (communicative) speech processes (Pouw, de Nooijer, van Gog, Zwaan, & Paas, 2014; Pouw & Hostetter, 2016).

Thus, just like co-speech gestures, co-thought gestures seem to provide the gesturer resources to think with. These resources cannot be directly associated with (communicative) speech processing, as co-thought gestures do not co-occur with speech or communicative intent. In the embedded/extended account of gesturing, Pouw et al. (2014; see also Pouw, Van Gog, & Paas, 2014) incorporated findings on co-speech and co-thought gesture effects on problem solving to propose an alternative cognitive function for gestures. The central tenet of this account is that gestures produce stable visual and proprioceptive sensory consequences that can be deployed to assist thinking. Gestures, as external bodily movements, embed and extend internal cognitive resources, which allow the cognitive system to solve problems in new or improved ways. For example, with regard to the co-thought gestures used in the mental rotation task of Chu and Kita (2008, 2011, 2016), an embedded/extended account of gesturing suggests that these gestures produced kinematic regularities (normally co-occurring with actually rotating an object) that can be used to predict the actual consequences of such a rotation. Such visual and proprioceptive information inherent to the gesture is not yet available internally, or more costly to internally simulate, and, therefore, the gesture aids in solving the mental rotation problem (see also Pouw & Hostetter, 2016).

A central prediction of the embedded/extended account of gesturing is that gestures reduce cognitive load by providing an (partial) external representation of the problem. Through this mechanism, gesturing can facilitate problem solving, compared to when only relying on limited capacity internal cognitive means (e.g., working-memory processes; mental-imagery processes). This idea aligns with a number of findings on co-speech gestures (see Pouw et al., 2014 for a review). Speakers who have a lower as opposed to higher visual working-memory capacity are more likely to use gestures when they talk (Chu, Meyer, Foulkes, & Kita, 2013). When speakers are confronted with complex compared to visually distracting information when telling a story they are more likely to gesture (Smithson & Nicoladis, 2014). In addition, gesturing during explanation of a math problem alleviates cognitive load, but only for those gesturers who have a relatively lower verbal working-memory capacity (e.g., Marstaller & Burianová, 2013). In sum, it seems that when cognitive load is high, either imposed by the complexity of the task, or by limited internal cognitive resources to solve the problem, gestures are more likely to occur and more likely to be supportive for cognitive processing.

Although this prediction fits research on co-speech gestures, it remains unclear whether co-thought gestures are more likely to occur and be effective when internal cognitive means are limited. More precisely, although there is some evidence that co-thought gestures are more likely to arise when task complexity is high (e.g., Chu & Kita, 2008; Logan et al., 2014), it is not clear yet how they relate to internal cognitive capacities such as working-memory capacity. Furthermore, in contrast to co-speech gestures (e.g., Hostetter & Alibali, 2008), it is still unknown under which conditions co-thought gestures arise (Chu & Kita, 2016).

### The present study

We studied the role of co-thought gesture during problem solving of the Tower of Hanoi (TOH), which has been previously used in gesture research (e.g., Trofatter, Kontra, Beilock, & Goldin-Meadow, 2014). Specifically, we compared the effects of instructed and spontaneous gesturing to ‘spontaneous’ non-gesturing (i.e., no explicit inhibition of gesture is taking place). This particular manipulation was chosen to exclude an alternative explanation for the benefits of gesturing compared to non-gesturing in prior research, namely an inhibition effect. Using a spontaneous non-gesturing group, a positive effect of gesturing compared to non-gesturing (when observed) is more likely to be due to the production of gesture, rather than due to a negative effect of having to inhibit automatic gesture production (e.g., Chu & Kita, 2011, Exp. 2 & 3; Cook, Yip, & Goldin-Meadow, 2012; Wagner, Nusbaum, & Goldin-Meadow, 2004). According to the embedded/extended account, an effect of producing gestures, as well as the spontaneous usage of gestures, is most likely to arise, and to positively affect problem-solving performance when cognitive load is high.

Performance on the TOH is positively and substantially correlated with visual and spatial working-memory capacity (Zook, Davalos, Delosh, & Davis, 2004), but it is unaffected by verbal working-memory capacity (Handley, Capon, Copp & Harper, 2002). Furthermore, visual working-memory capacity measures have been found to predict co-speech gesture frequency (Chu et al., 2013). Thus, both visual and spatial working-memory performance, as a proxy for visual and spatial mental-imagery ability, respectively, were assessed to test our assumptions regarding cognitive load in these tasks. This allowed us to identify which internal cognitive processes gestures may support or replace (in the current tasks).

We hypothesized that (1) participants would produce more co-thought gestures under high cognitive load conditions as determined by task complexity (i.e., higher complexity = higher load), visual and spatial mental-imagery ability (lower capacity = higher load), and the combination of both (gesture-likelihood hypothesis); (2) co-thought gesturing (spontaneous and instructed) should positively affect mental problem-solving performance (as evidenced by speed and efficiency of subsequent actual performance) in comparison to non-gesturing, under high cognitive load conditions as determined by task complexity (i.e., higher complexity = higher load), visual and spatial mental-imagery ability (lower capacity = higher load), and the combination of both (gesture-effect hypothesis).

## Method

### Participants and design

A total of 76 Dutch university students participated in this study in partial fulfillment of course requirements or for a financial compensation of 7.50 euros (about 8.30 USD). One participant was excluded due to a technical malfunction in the task presentation and two additional participants were excluded, because they did not comply with the gesture instruction. This resulted in a total sample of 73 (41.1% men, *M*_age_ = 20.60, SD = 2.06, range 18–31 years). Participants were randomly assigned to one of the two conditions (gesture-instructed vs. gesture-allowed). Nine participants said to have played the TOH in the past, but they were equally divided across conditions, *χ*^2^(1) = 0.123, *p* = .725. This experiment was performed in accordance with the guidelines of the ethical committee of the Department of Psychology, Education, and Child Studies, at the Erasmus University Rotterdam.

### Materials

#### Visual working-memory capacity: visual pattern test

Visual working-memory capacity was measured with an adapted version of the Visual Pattern Test (original VPT; Della Sala, Gray, Baddeley, & Wilson, 1997; adapted VPT; developed and kindly provided to us by Chu et al., 2013). Participants were shown a matrix, in various patterns, wherein half of the cells (i.e., squares of 15 mm × 15 mm) were colored black. Each pattern was displayed for 3 s, after which all the squares turned white and each square was labeled with one unique letter. Participants indicated the pattern of black squares by naming the letters of the corresponding squares aloud in a non-specific order. The VPT consisted of 25 trials, with blocks of five trials per difficulty (from seven to 11 black squares). Before the start of the task, participants were provided with two practice trials (3 and 4 black squares, respectively). If participants failed to recall all the letters, it was scored as an incorrect response. After five consecutive incorrect responses within one block of trials, the experimenter ceased the task.

#### Spatial working-memory capacity: Corsi block task

Spatial working-memory capacity was measured with the Corsi block task (CBT; Corsi, 1972), as used by Chu et al., (2013). The task consisted of four blocks, each containing of five trials. In each trial, nine empty irregularly placed squares (15 mm × 15 mm) were displayed on the computer screen. One square at a time turned black for 1 s, with an inter-stimulus interval of 500 ms between each transition. The first block consisted of a sequence of five squares turning black, with each subsequent difficulty level adding one black square to the sequence (the fourth block sequence thus consisted of eight squares turning black). After the last square in the sequence turned black, a letter appeared in the center of each square. Participants verbally repeated the letters in the correct order, following the order of the squares turning black. If the participants failed to recall all the letters in the correct order, the trial was scored as an incorrect response. After five incorrect responses in one block of trials, the experimenter ceased the task.

#### Tower of Hanoi

The Tower of Hanoi (TOH) consisted of a wooden structure with a rectangular base (17 cm tall, 50 cm wide, and 1 cm deep) with three evenly spaced pegs (13.5 cm tall, 1 cm in diameter) mounted on top. The ‘easy’ version of the task consisted of three wooden discs (size disc 1: 7.5 cm in diameter; size disc 2: 7 cm in diameter; size disc 3: 6.5 cm in diameter) and the more complex version of the task of four discs (size disc 4: 6 cm); all discs were 1 cm in height. They were initially stacked on the left most peg and could be placed on the other pegs during the problem-solving process. When participants took longer than 300 s to solve either of the two trials, the trial would be aborted.

#### Self-report questions

##### Mental effort, difficulty, and interest

Mental effort, perceived difficulty, and experienced interest were obtained via self-reports after each problem-solving trial, because effort and difficulty should be affected by task difficulty, whereas interest is assessed to check that this would not differ (and, therefore, will not explain hypothesized differences) among groups. Participants had to respond on a five-point rating scale (see Paas, Tuovinen, Tabbers, & Van Gerven, 2003): “How much mental effort did you exert during the task?” (mental effort; 1 = ‘very low mental effort’ to 5 = ‘very high mental effort’), “How difficult did you find this task” (difficulty; 1 = ‘not difficult’ to 5 = ‘very difficult’), and “How interesting did you find this task” (interest; 1 = ‘not interesting’ to 5 = ‘very interesting’).

### Procedure

Before the start of the experiment, participants consented to being video-recorded during the experiment. Participants were tested individually with the experimenter present in the room (but they could not see the experimenter, while they were working on the tasks). The order of the working-memory capacity tasks was counterbalanced, such that, in both conditions, half of the participants started with the spatial working-memory task (CBT), whereas the other half would start with the visual working-memory task (VPT).

After participants completed both working-memory capacity tasks, the experimenter started the instructions for the TOH. First, they were told about the end goal: getting the arrangement of discs on the outer left peg on the outer right peg, in the same order. Then, they were told about the two constraints: (I) only one disc at a time may be moved from one peg to another and (II) a larger disc cannot be placed on a peg that already contains a smaller disc. After each instruction, the experimenter verified whether subjects understood the instructions by asking them to verbally repeat the rules. Participants were informed that before solving the TOH as fast as possible, they would mentally plan the moves without manipulating the TOH for 150 s (the TOH was placed just outside arms’ reach: 90 cm from the participant).

Participants told that they should find and rehearse the correct moves repeatedly during this phase. Half of the participants were additionally instructed “to gesture, in other words, to think with your hands during this mental planning phase in a way that suits you” (gesture-instructed condition). During this instruction, the experimenter also performed a quick demonstration of a typical index pointing gesture, which consisted of pointing movements directed at the TOH pegs. Thus participants were cued to use pointing gestures in the instructed gesture condition, but could gesture in any way that suited them. The other half of the participants did not receive any gesture instructions (gesture-allowed condition). Participants first solved the easier 3-disc TOH, after which they reported mental effort invested in the task, perceived task difficulty, and their interest in the task. Subsequently, the same routine was repeated (i.e., mental problem-solving phase followed by physical solving phase) for the more complex 4-disc TOH. After the experiment, participants filled out a questionnaire including demographic questions (age, sex, study program, and prior experience with the task) and questions about what they thought was the purpose of the experiment. Finally, participants were debriefed and thanked for their participation.

### Scoring and data analysis

#### Visual pattern test

The final score was the proportion of the correct responses out of all 25 trials (higher proportion approximates higher visual working-memory capacity).

#### Corsi block task

The final score was the proportion of the correct responses out of all 20 trials (higher proportion approximates higher spatial visual working-memory capacity).

#### Tower of Hanoi

For each of the two problem-solving trials, we obtained solution time and number of solving steps, with faster solving times and lower number of solving steps reflecting a higher performance. For the 3-disc TOH and the 4-disc TOH, the minimal amount of steps necessary to solve the task was 7 and 14 steps, respectively. A step was counted when participants placed a disc on another peg (so not on the same peg) once the disc was released (i.e., if they changed their mind before releasing the disc, this was not counted as a step).

#### Gesture

Videos were coded for the gesture group for each TOH problem. The participants in the gesture-allowed group were re-divided into two gesture categories of (non-gesturing vs. spontaneous gesturing), and because all participants in the instructed gesturing gestured, they were all assigned to the instructed gesturing group. This means that three gesture groups could be distinguished for each TOH task: (1) non-gesturing, (2) spontaneous gesturing, and (3) instructed gesturing.

**Fig. 1:**
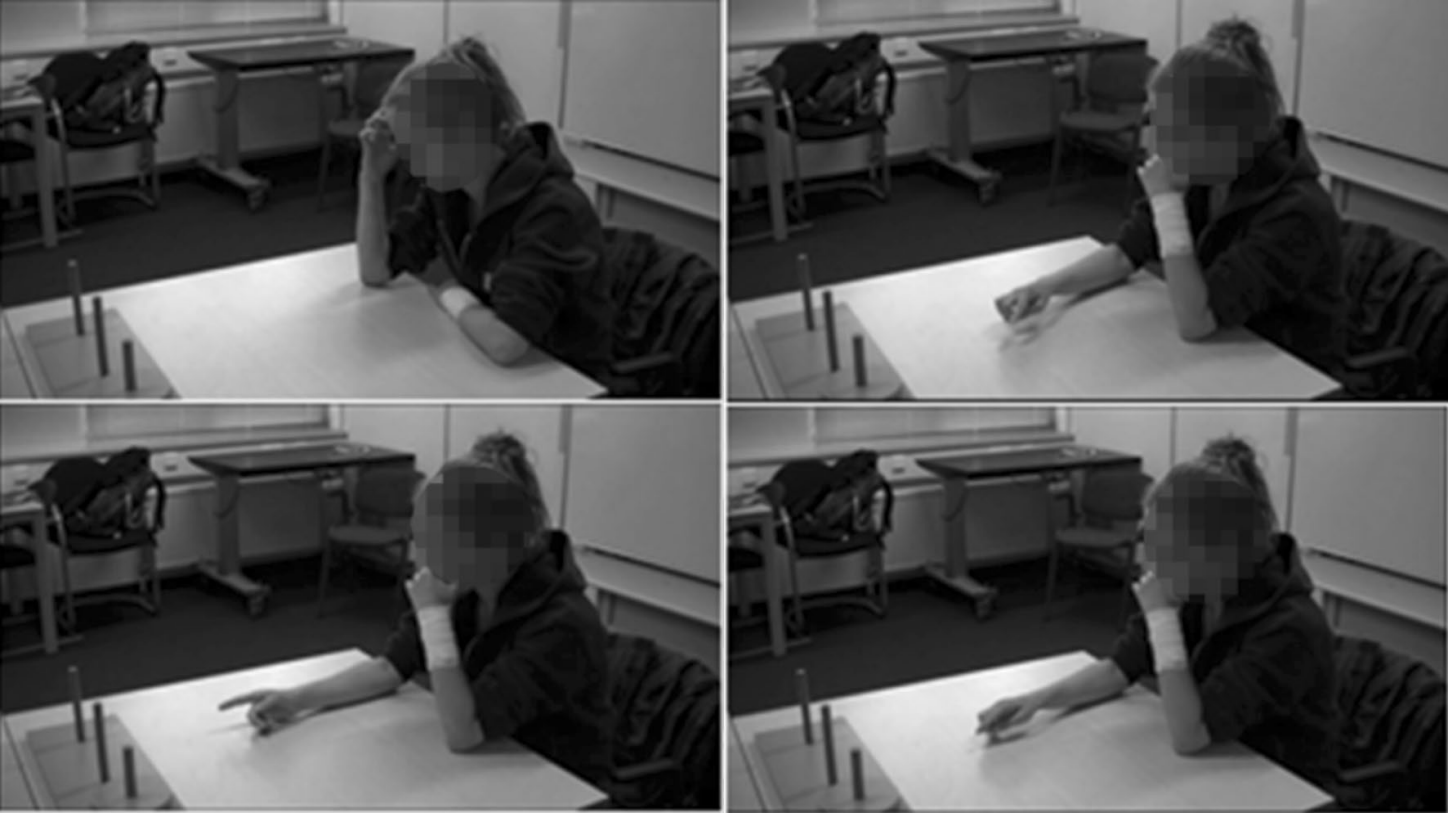
Examples of spontaneous gestures arising during the mental preparation phase prior to solving the TOH

In addition, we looked at form (pointing vs. iconic) and frequency of gesture. Almost all instructed and spontaneous gesturing consisted of pointing gestures (see Fig. 1 for an example) with three exceptions. Two participants briefly used counting gestures, and one participant briefly used iconic gestures with two hands simulating picking up and moving all the discs. Given the practical absence of iconic gestures, as a measure of gesture frequency, we only counted the number of pointing gestures; in such a way that each rest point after a pointing gesture (either whole hand- or finger-pointing) was considered as one instance. A research assistant counted all the gestures, and the first author independently counted 10.4% of the sample to check for reliability. A high degree of reliability was found between both counts; the average intra class correlation coefficient was 0.991 with a 95% confidence interval from 0.910 to 0.996, *p* = .001.

#### Data analyses

An annotated script is provided in the supplementary materials, including all the data-processing procedures and raw data, and all analyses provided online via: https://osf.io/fydsv/. Because solution time for the 3-disc (but not the 4-disc) task was heavily skewed, we logarithmically transformed this variable. We also logarithmically transformed number of solving steps for the 3-disc task. This resulted in improved skewness and kurtosis scores for 3-disc solution time (skewness = 1.99, SE = 0.28, kurtosis = 6.40, SE = 0.56), but did not completely resolve deviances from normality for the 3-disc solving steps (skewness = 2.73, SE = 0.28, kurtosis = 7.09, SE = 0.56) and the 4-disc solving steps (skewness = − 2.49, SE = 0.28, kurtosis = 8.64, SE = 0.56). Thus, solution times for the TOH 3-disc and TOH 4-disc have acceptable deviances from normality without (4-disc) or after log transformation (3-disc), while inferences from solving steps measures should be treated with caution. During the 4-disc TOH, one participant forgot to follow the rules and made several mistakes during the 4-disc TOH (and was, therefore, excluded from analysis of the 4-disc TOH). Solving steps on the 4-disc was not normally distributed and we, therefore, used a log transformation.

## Results

### Overall performance TOH

Overall mean solution time and solving steps for the 3-disc and 4-disc TOH can be obtained from Table 1. In terms of solving steps, most participants had a ceiling performance on the TOH 3-disc task (82.2% for the 3-disc TOH and 37.7% for the 4-disc TOH).

**Table 1:** Means, standard deviations, and correlations of VPT, CBT, number of steps, and solution time on the tower of hanoi 3-disc and 4-disc

	Mean	SD	Skew	Kurtosis	1	2	3	4	5
1. VPT score	0.50	0.21	− 0.30	− 0.96					
2. CBT score	0.41	0.15	0.03	− 0.22	0.36**				
3. Solution time TOH 3 (in seconds)	20.52	16.00	4.87	27.23	− 0.19	− 0.16			
4. Solution time TOH 4 (in seconds)	84.61	57.83	0.90	− 0.36	− 0.57**	− 0.41**	0.21		
5. Number of steps TOH 3	7.63	1.65	3.12	9.63	0.00	− 0.14	0.74**	0.09	
6. Number of steps TOH 4	23.39	11.12	1.54	2.05	− 0.43**	− 0.30*	0.18	0.88**	0.15

*p < .05, **p < .01

### Visual and spatial working-memory capacity

Next, we checked for unintended differences in working-memory measures between conditions. We did not find statistically significant differences between the gesture-allowed and -instructed condition in the proportion correct trials on the VPT, *t*(71) = − 0.49, *p* = .477 or CBT, *t*(70) = 0.33, *p* = .744 (see Table 1). The VPT and CBT did not correlate with solution time on the 3-disc. Both the VPT and CBT were negatively correlated with solution time on the 4-disc TOH, such that higher VPT and CBT scores were associated with faster solution times and a lower amount of solving steps.

### Gesture-likelihood hypothesis

On the 3-disc TOH, 28.9% (*N* = 11/38) participants spontaneously gestured during the mental solving phase. On the 4-disc TOH, 47.3% (*N* = 18/38) of the participants in the gesture-allowed condition spontaneously gestured during the mental solving phase. Table 2 displays the average gesture frequency for each gesture group. The gesture-likelihood hypothesis predicted that the percentage of spontaneous gesturing participants (non-gesturing vs. spontaneous gesturing) and frequency (number of gestures) would be higher in the complex task and for those with a lower visual and/or spatial working-memory capacity. The subsequent analyses tested that hypothesis and also provided a manipulation check on whether participants who were instructed to gesture, indeed do so more often in terms of gesture frequency as compared to those who spontaneously gestured.

**Table 2:** Means, standard deviations of the VPT, CBT, number of steps, solution time, and gesture frequency on the tower of Hanoi 3-disc and 4-disc for each gesture group

	Gesture group		
	Non-gesturing (*N* = 27)	Spontaneous gesturing (*N* = 11)	Instructed gesturing (*N* = 35)
	Mean (SD)	Mean (SD)	Mean (SD)
TOH 3			
VPT	0.48 (0.20)	0.51 (0.20)	0.51 (0.23)
CBT	0.42 (0.17)	0.42 (0.14)	0.41 (0.15)
Solution time	23.37 (24.56)	19.82 (8.05)	18.54 (7.15)
Number of steps	7.63 (1.78)	7.55 (0.93)	7.66 (1.77)
Gesture frequency	0.00 (0.00)	23.45 (13.87)	47.70 (25.78)
Difficulty	1.81 (0.74)	1.91 (0.94)	1.85 (0.61)
Mental effort	2.04 (0.94)	2.27 (1.19)	2.06 (0.81)
Interest	2.85 (0.95)	3.27 (0.79)	3.00 (0.85)

	Non-gesturing (*N* = 19)	Spontaneous gesturing (*N* = 18)	Instructed gesturing (*N* = 35)
	Mean (SD)	Mean (SD)	Mean (SD)
TOH 4			
VPT	0.45 (0.20)	0.52 (0.21)	0.51 (0.22)
CBT	0.42 (0.18)	0.42 (0.15)	0.41 (0.15)
Solution time	103.00 (65.27)	70.94 (54.35)	80.09 (53.55)
Number of steps	24.15 (9.76)	23.65 (13.00)	22.79 (11.18)
Gesture frequency	0.00 (0.00)	32.78 (25.92)	66.20 (18.59)
Difficulty	3.30 (0.80)	3.71 (0.85)	3.49 (0.74)
Mental effort	3.25 (0.72)	3.76 (0.83)	3.69 (0.87)
Interest	3.40 (0.94)	3.94 (0.56)	3.71 (0.71)

#### Spontaneous gesture likelihood

To assess whether there was a significant difference in the percentage of spontaneously gesturing participants in the gesture-allowed condition across complexity, we performed a dependent Chi-square test (McNemar Change Test), which revealed that spontaneous gesturing was, indeed, more likely in the 4-disc TOH than in the 3-disc TOH, *χ*^2^(1) = 4.00, *p* = .039.

However, those participants who gestured spontaneously during the mental solving phase of the solution procedure for the 3-disc TOH, did not differ from non-gesturing participants with regard to VPT score, *t*(36) = − 0.35, *p* = .728. Similarly, participants who gestured spontaneously during mental solving phase of the 4-disc TOH did not differ from non-gesturing participants with regard to VPT score, *t*(34) = − 1.87, *p* = .071. This was also the case for spatial working memory, during the 3-disc TOH, spontaneous gesturing participants did not reliably differ from non-gesturing participants with regard to CBT score, *t*(35) = − 0.09, *p* = .962, nor for the 4-disc TOH, *t*(33) =− 0.31, *p* = .762. Thus, in contrast to our predictions, visual (VPT) and spatial (CBT) working-memory capacity did not affect whether participants gestured or not (i.e., gesture group).

#### Spontaneous and instructed gesture frequency

For these analyses, participants in the gesture-allowed condition were assigned to the spontaneous gesture group when they gestured during either the 3-disc or 4-disc TOH. First, a repeated-measures ANOVA was conducted to assess whether task complexity (within-subjects: 3-disc TOH vs. 4-disc TOH) and gesture group (between-subjects: spontaneous vs. instructed gesture) predicted gesture frequency. In a second analysis, VPT score was included as a covariate to test whether visual working memory co-varied with gesture frequency. The first analysis yielded a significant main effect of task complexity, *F*(1, 52) = 26.62, *p* < .001, *η*_*p*_^2^ = 0.339, and gesture group, *F*(1, 52) = 43.05, *p* < .001, *η*_*p*_^2^ = 0.453, but no interaction between complexity and gesture group, *F*(1,52) = 0.02, *p* = .882. These results indicate that gesture frequency was significantly lower during the mental solving phase for the 3-disc TOH than for the 4-disc TOH and that participants who were instructed to gesture did so more often than participants who spontaneously gestured.

Results of the ANCOVA showed that performance on the VPT did not account for variance in gesture frequency, *F*(1, 51) = 0.34, *p* = .562, and the effect of complexity on gesture frequency dissipated when including VPT score as covariate, *F*(1, 51) = 0.22, *p* = .641. There were no interactions of VPT with complexity, *F*(1, 51) = 3.27, *p* = .077, or gesture group *F*(1, 51) = 0.20, *p* = .887. When entering CBT as a covariate this yielded similar non-significant results, *F*(1, 50) = 0.32, *p* = .574. Again, no statistically significant interactions were obtained for CBT across complexity or gesture type, *F*(1, 50) = 0.01, *p* = .924. These findings suggest that visual and spatial working-memory capacities did not predict gesture frequency (either when instructed to do so or when spontaneously produced).

### Gesture-effect hypothesis

The gesture-effect hypothesis predicted that co-thought gesturing (spontaneous and instructed) would positively affect problem-solving performance (as evidenced by solution time of subsequent actual performance) under high cognitive load conditions in comparison to participants who did not gesture but were allowed to (i.e., ‘spontaneous’ non-gesturing). We first analyzed whether VPT, gesture group (non-gesturing vs. spontaneous gesturing vs. instructed gesturing) and their interaction affected solution time on the 3-disc and 4-disc TOH in two multiple stepwise hierarchical regression analyses (i.e., separate analysis for each task complexity level). Note that the gesture-effect hypothesis predicts interaction effects—especially on the more complex task (i.e., solution time 4-disc TOH)—of VPT and gesture group, such that those with lower VPT scores improve in performance when using gestures (i.e., instructed or spontaneous) as compared to those who did not gesture and who have similar VPT scores.

We entered VPT (mean centered) in the first step, and the dummy variables for spontaneous gesturing (0 = non-gesturing; 1 = spontaneous gesturing) and instructed gesture (0 = non-gesturing; 1 = instructed gesturing) in the second step. In the third step, we entered two interaction terms of the centered VPT with the instructed and spontaneous gesture group dummy variables. As can be seen in Table 3, no significant predictors were obtained for solution time on the 3-disc TOH. However, on the 4-disc TOH, there was a main effect of VPT on solution time, which remained so in the final model, *t*(68) = − 5.44, *p* < .001, *partial r* = − .570. There were no main effects of spontaneous gesturing, *t*(68) = − 0.92, *p* = .361, or instructed gesturing, *t*(68) = − 0.31, *p* = .758. However, in the final model (explaining 37% of the variance), the interaction terms of VPT and spontaneous gesturing, *t*(68) = 2.88, *p* = .005, *partial R* = .341, as well as instructed gesture gesturing, *t*(68) = 2.42, *p* = .018, *partial R* = .292, were significant in the predicted direction. Specifically, spontaneous and instructed gestures positively affected performance (i.e., less time needed to solve the task) on the task as compared to non-gesturing, especially when participants had a lower VPT score.

**Table 3:** Standard beta values and significance levels of the final step of the hierarchical regression analyses, in which solution time (log transformed) and TOH4 was predicted from vpt score and gesture group (spontaneous gesturing and instructed gesturing) and the respective interaction terms

	Three-disc TOH (*N* = 73)			Four-disc TOH (*N* = 72)		
	Beta	*t*	Sig	Beta	*t*	Sig
Constant		35.46	0.000		8.69	0.000
VPT	− 0.44	− 2.22	0.030	− 0.99	− 5.44	0.000
Spontaneous gesturing	0.02	0.17	0.865	− 0.13	− 1.11	0.273
Instructed gesturing	− 0.05	− 0.05	0.678	− 0.05	− 0.45	0.653
VPT × spontaneous gesturing	− 0.01	− 0.13	0.926	0.36	2.88	0.005
VPT × instructed gesturing	0.31	0.31	0.098	0.40	2.42	0.018

**Fig. 2:**
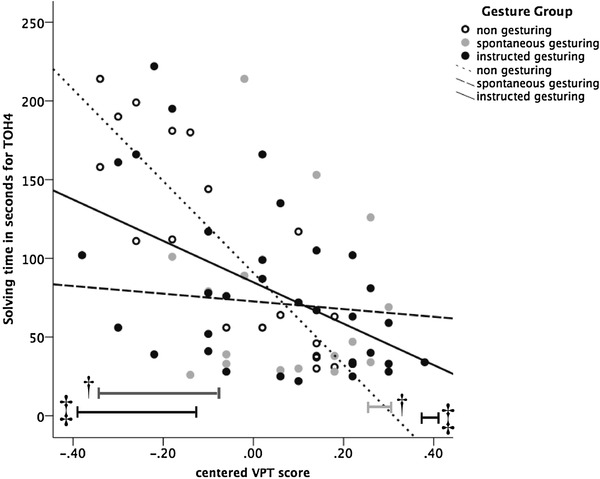
Regression slopes for each gesture group (non-gesture vs. spontaneous gesturing vs. instructed gesturing) on amount of time in seconds necessary to solve the problem on the difficult 4-disc TOH, with centered VPT score on the horizontal axis. The “**†**” denotes the region where the effect of spontaneous gesture prevalence (vs. no gesture) was significant. The “**‡**” denotes the region of significance for instructed gesture prevalence

To further explore these interactions, we performed Johnson–Neyman Regions of Significance Tests using PROCESS (Hayes, 2013) to assess the nature of the two interaction effects of instructed and spontaneous gestures with the VPT (see Fig. 2). For participants with a centered VPT score below − 0.13 (26.4% of the sample), there was a significant positive effect of instructed gesturing on solution time on the 4-disc TOH, range *t*(52) = − 2.63 to − 2.01, range *p* = .011:0.050. However, results further showed that those with a centered VPT score higher than 0.36 (1.89% of the sample) seemed to be slowed down by instructed gesturing according to the model, range *t*(49) = 2.01 to − 2.16, range *p* = .035:0.050.

Similarly, in the second analysis where only spontaneous gesturing is compared to ‘spontaneous’ non-gesturing (coded 0 = non-gesturing, 1 = spontaneous gesturing), participants with a centered VPT score lower than − 0.083 (33.3% of the sample) profited from gesturing, range *t*(32) = − 2.93 to − 2.04, range *p* = .050:0.006. In addition, participants with a centered VPT score higher than 0.26 (2.8% of the sample) seemed to be slowed down by spontaneous gesturing, range *t*(49) = 2.01 to − 2.16, range *p* = .050:0.035.

We also ran the previous hierarchical regression analyses with the CBT (reported in Table 4), but no statistically significant interactions of CBT and instructed or spontaneous gesturing were obtained in explaining solution time on the 3-disc TOH. This was also the case for solution time on the 4-disc TOH.

**Table 4:** Standard beta values and significance levels of the final step of the hierarchical regression analyses, in which solution time (log transformed) for the TOH4 was predicted from cbt score and gesture group (spontaneous gesturing and instructed gesturing dummy’s) and the respective interaction terms

	Three-disc TOH (*N* = 73)			Four-disc TOH (*N* = 72)		
	Beta	*t*	Sig	Beta	*t*	Sig
Constant		35.00	0.000		8.74	0.000
CBT	− 0.18	− 1.00	0.321	− 0.41	− 2.24	0.028
Spontaneous gesturing	0.01	0.05	0.962	− 0.22	− 1.67	0.100
Instructed gesturing	− 0.08	− 0.63	0.534	− 0.21	− 1.61	0.112
CBT × spontaneous gesturing	− 0.02	− 0.14	0.888	0.03	0.25	0.803
CBT × instructed gesturing	− 0.05	− 0.30	0.764	− 0.02	− 0.09	0.927

### Mental effort, difficulty, and interest

To assess whether task complexity and gesture group affected self-report measures of invested mental effort, experienced difficulty, and interest, three separate 2 (Task Difficulty: TOH3 and TOH4) × 3 (gesture group: non-gesturing, spontaneous gesturing, and instructed gesturing) repeated-measures ANOVAs were conducted (Bonferroni correct, *α* = 0.017) (see Table 2 for means and SDs for self-report ratings across different gesture groups). Participants reported higher mental effort, *F*(1,68) = 172.44, *p* = .000, *η*_*p*_^2^ = 0.717, difficulty, *F*(1,68) = 244.18, *p* = .000, *η*_*p*_^2^ = 0.782, and interest, *F*(1,68) = 75.97, *p* = .000, *η*_*p*_^2^ = 0.528, on the 4-disc TOH compared to the 3-disc TOH. The analyses yielded no effects of gesture group on mental effort, *F*(1,68) = 1.29, *p* = .283, task difficulty, *F*(1,68) = 0.61, *p* = .549, and interest, *F*(1,68) = 2.62, *p* = .080, nor were there any interactions with task complexity, respectively, *F*(1,68) = 0.85, *p* = .433, *F*(1,68) = 0.40, *p* = .672, *F*(1,68) = 0.55, *p* = .577.

## Discussion

We hypothesized that gestures are more likely to arise in (gesture-likelihood hypothesis) and positively affect (gesture-effect hypothesis) problem solving under conditions of higher cognitive load (i.e., higher task complexity, lower visual, or spatial working-memory capacity).

### Gesture-effect hypothesis

In line with the gesture-effect hypothesis, our results indicate that participants who gestured, either spontaneously or instructed, subsequently performed the task faster than participants who did not gesture of their own accord, but only for those with a lower visual working-memory capacity. Interestingly, this interaction effect was not found for spatial working-memory capacity.

The current novel findings on gesture’s effect on problem solving offer additional support to the general idea that gesturing may be especially effective when internal cognitive resources are limited (Pouw et al., 2014). Furthermore, gestures seem to provide resources to support, or counteract, limited *visual* rather than *spatial* working-memory processes in the current task. Before addressing this interesting unexpected difference between visual and spatial working-memory capacity, we should address how gestures might have benefited problem solving in the current task. We argue that, when confronted with mentally exploring the solution space of the Tower of Hanoi, participants simulate the transformations of the discs/pieces from one place to another through mental imagery. Such mental-imagery processes are likely to work in concert with the visual information that is provided by the static presentation of the task setup, allowing simulated moves to be projected on the pegs (Kirsh, 1995). Simulating the moves also entails continuously memorizing the positions of the discs/pieces that are moved during the mental simulation. Keeping track of simulated moves, therefore, requires visual working-memory processes, and those that have lower capacities are more likely to lose track of the changing positions of the discs/pieces during their more unstable visual imaginations. We argue that producing gestures offer stable visual and proprioceptive information regarding the hands in space, which allow a way to spatially index or “temporarily” locking simulated moves represented by the hand in space, thereby alleviating the relatively unstable visual imagery processes that would, otherwise, fulfill this tracking function. Indeed, in a companion study, we have found evidence that when participants with a lower visual working-memory capacity are instructed to gesture (or not to gesture) during mental preparation of the TOH, they produce less (or more) eye movements, suggesting that moving the hands allows for a way to stabilize simulations in a less visually demanding way (Pouw, Mavilidi, Van Gog, & Paas, 2016).

The finding that spatial working-memory capacity did not interact with an effect of gesture on performance was surprising, and can only be met with substantial speculation. A superficial explanation would be that the pointing gestures in the current case are more potent for keeping active (i.e., locking) the visuo-spatial static positions of the multiple pieces throughout the mental simulation rather than to keep track of the spatial sequential trajectories of the simulated discs. Yet, this explanation begs the question why pointing gestures are potent in only this way. After all, these pointing gestures do contain information about spatial sequential trajectories. In sum, the current findings cannot provide insight into why we find this interesting difference. However, an interesting alignment with the current results is that visual working-memory capacity has been found to be more predictive for co-speech gesture frequency than spatial working-memory capacity (Kita et al., 2017), corroborating the idea that gestures may be especially potent to alleviate limitations in visual working-memory capacity.

There are some limitations that should caution us about over-interpreting the evidence in favor of the gesture-effect hypothesis. For instance, one could argue that the current gesture effects arose, because gestures reflect efficient mental simulations rather than contribute to them. However, we think that the present findings do not provide much support for this possibility. If only spontaneous (and not instructed) gesturing would have affected performance, it could be argued that gestures are a consequence of effective mental planning rather than having an assisting function. However, the gesture manipulation in this study through instruction showed similar positive gesture effects as spontaneous gestures. This implies that gesturing is in a causal relation with gesture production and performance, rather than mental post-cursor of cognitive performance (for a discussion, see Pouw et al., 2014).

It is possible that problem solvers utilized verbal strategies in solving the TOH problem. For example, they might label discs and positions as to come to a higher order understanding of the problem solution space. Although our research does not address this possibility, we doubt that verbal strategies were employed in the current study for several reasons. First, research on TOH does not find relations between verbal capacities and performance on the TOH (Handley et al., 2002). Second, it is well known that in spatial tasks such as these, mental imagery of the non-propositional kind is a pre-dominant strategy to solve such tasks competently. For example, there is an indication that mental abacus experts employ a visual rather than verbal strategy to perform complex calculations (Frank & Barner, 2012), and mental rotations are performed through imagining perceptual reenactments rather than abstract re-descriptions of the solution space (Chu & Kita, 2016; Shephard & Metzler, 1988; however also see Hugdahl, Thomsen & Ersland, 2006). Third, the development of a verbal strategy would likely emerge only after problem solvers have been fully familiarized with the solution space of the TOH. In the current study, participants performed a limited number of TOH trials, which makes it arguably less likely that they were able to solve the task verbally. Nevertheless, we cannot entirely rule out that silent verbalizing occurred. To rule that out, future research could measure laryngeal muscle activity during these tasks using magnetoencephalography, or use a concurrent verbal task that interferes with employing verbal strategies as to assess whether performance diminishes under such circumstances.

### Gesture-likelihood hypothesis

The results of this study provide only partial support for the gesture-likelihood hypothesis, dictating that gesticulation (in terms of gesture group or frequency) is more likely when cognitive load is high. The percentage of participants who spontaneously gestured increased along with task complexity. Moreover, gesturing was more frequent during the more complex TOH task. Note though, that even this partial support should be interpreted with some caution, because the higher the task complexity, the larger the number of steps needed to solve the task. Even though participants, indeed, experienced higher cognitive load (i.e., higher mental effort and difficulty ratings) in the more complex TOH task, the finding that gesture frequency was higher might be due to the fact that this task required more steps to solve as opposed to the cognitive load the task imposed. Of course, in the current tasks, it is difficult to manipulate complexity without (a) increasing the number of steps to solve the task or (b) keeping the task the same but adding a secondary task to manipulate cognitive load. Future research could resolve this issue by adopting option b in the design (see, e.g., Marstaller & Burianová, 2013).

Yet, critical points regarding the gesture-likelihood hypothesis aside, it should be emphasized that the current study shows that the present physical setup of the TOH, respectively, elicits spontaneous co-thought gestures. This is an important finding in and of itself, as it provides a paradigm for further investigation into natural occurrences of co-thought gesticulation, next to the few paradigms that are currently used (e.g., mental rotation and route learning). Moreover, the current findings hint towards future research that contrasts the cognitive function of co-thought with that of co-speech gestures. For example, the spontaneous gestures observed in the current TOH task were virtually all deictic (i.e., pointing) gestures, whereas the previous research has established that co-speech gestures in explaining solving the TOH are often iconic in nature (e.g., grasping movements; e.g., Cook & Tatenhaus, 2009; Trofatter et al., 2014). This begs the question whether the form (pointing vs. iconic) is connected with different functions relating to problem solving vs. speech processes (see, e.g., Cappuccio, Chu, & Kita, 2013; Chu & Kita, 2016). Note that it is possible that participants are more or less likely to produce particular kind of gesture (e.g., pointing vs. iconic gestures) or are more or less inclined to gesture if the task was within manipulable reach. Indeed, the previous research has shown that increasing the perceived manipulability of objects changes how likely participants are to gesture about those objects in mental rotation (Chu & Kita, 2016).

### Conclusion

The current findings offer novel evidence that next to co-speech gestures (e.g., Beilock & Goldin-Meadow, 2010), co-thought pointing gestures are spontaneously solicited when participants are confronted with a problem-solving task. Gestures seem to be especially productive for problem-solving performance when tasks are more complex (corroborating the previous findings, Chu & Kita, 2008), and interact with visual (but not spatial) working-memory capacity. Moreover, instructing others to do so may aid problem-solving performance (at least for adult problem solvers; see Pouw, Van Gog, Zwaan, Agostinho, & Paas, 2018). The present study, therefore, provides an additional support that gestures’ cognitive function may go beyond speech processing and provides the initial insight into improving educational environments by soliciting embodied problem solving. For instance, from the results, we can imagine that the current learning procedures for tasks with a heavy working-memory demand can be more accommodating for beginning learners (as they experience a higher complexity of the task) when these learners are prompted to think out their moves with their hands.

## References

[CR1] Alibali MW, Spencer RC, Knox L, Kita S (2011). Spontaneous gestures influence strategy choices in problem solving. Psychological Science.

[CR2] Beilock SL, Goldin-Meadow S (2010). Gesture changes thought by grounding it in action. Psychological Science.

[CR3] Brooks, N. B. (2014). *Movements for doing, thinking, and speaking: The relationship between motor experience and gesture in novice mental abacus users* (Doctoral dissertation, the University of Chicago).

[CR4] Cappuccio ML, Chu M, Kita S (2013). Pointing as an instrumental gesture: Gaze representation through indication. Humana.Mente: Journal of Philosophical Studies.

[CR5] Chu M, Kita S (2008). Spontaneous gestures during mental rotation tasks: Insights into the microdevelopment of the motor strategy. Journal of Experimental Psychology: General.

[CR6] Chu M, Kita S (2011). The nature of gestures’ beneficial role in spatial problem solving. Journal of Experimental Psychology: General.

[CR7] Chu M, Kita S (2016). Co-thought and co-speech gestures are generated by the same action generation process. Journal of Experimental Psychology: Learning, Memory, and Cognition.

[CR8] Chu M, Meyer A, Foulkes L, Kita S (2013). Individual differences in frequency and salience of speech-accompanying gestures: the role of cognitive abilities and empathy. Journal of Experimental Psychology: General.

[CR9] Cook SW, Tatenhaus MK (2009). Embodied communication: Speakers’ gestures affect listeners’ actions. Cognition.

[CR10] Cook SW, Yip TK, Goldin-Meadow S (2012). Gestures, but not meaningless movements, lighten working memory load when explaining math. Language and Cognitive Processes.

[CR11] Delgado B, Gómez JC, Sarriá E (2011). Pointing gestures as a cognitive tool in young children: experimental evidence. Journal of Experimental Child Psychology.

[CR12] Della Sala S, Gray C, Baddeley A, Wilson L (1997). The visual patterns test: A new test of short-term visual recall.

[CR13] Frank MC, Barner D (2012). Representing exact number visually using mental abacus. Journal of Experimental Psychology: General.

[CR14] Garber P, Goldin-Meadow S (2002). Gesture offers insight into problem-solving in adults and children. Cognitive Science.

[CR15] Goldin-Meadow S, Nusbaum H, Kelly SD, Wagner S (2001). Explaining math: Gesturing lightens the load. Psychological Science.

[CR16] Handley SJ, Capon A, Copp C, Harper C (2002). Conditional reasoning and the Tower of Hanoi: The role of spatial and verbal working memory. British Journal of Psychology.

[CR17] Hayes AF (2013). *Introduction to mediation, moderation, and conditional process analysis: A regression*-*based approach*.

[CR19] Hostetter AB, Alibali MW (2008). Visible embodiment: Gestures as simulated action. Psychonomic Bulletin & Review.

[CR20] Hugdahl K, Thomsen T, Ersland L (2006). Sex differences in visuo-spatial processing: an fMRI study of mental rotation. Neuropsychologia.

[CR21] Kirsh, D. (1995). Complementary strategies: Why we use our hands when we think. In *Proceedings of the seventeenth annual conference of the cognitive science society* (pp. 212–217).

[CR22] Kita S, Alibali MW, Chu M (2017). How do gestures influence thinking and speaking? The gesture-for-conceptualization hypothesis. Psychological Review.

[CR23] Logan T, Lowrie T, Diezmann CM (2014). Co-thought gestures: Supporting students to successfully navigate map tasks. Educational Studies in Mathematics.

[CR24] Macken L, Ginns P (2014). Pointing and tracing gestures may enhance anatomy and physiology learning. Medical teacher.

[CR25] Marstaller L, Burianová H (2013). Individual differences in the gesture effect on working memory. Psychonomic Bulletin Review.

[CR26] Paas F, Tuovinen JE, Tabbers H, Van Gerven PW (2003). Cognitive load measurement as a means to advance cognitive load theory. Educational Psychologist.

[CR27] Pouw W, Hostetter A (2016). Gesture as predictive action. Reti, Saperi, Linguaggi: Italian Journal of Cognitive Sciences.

[CR28] Pouw WT, Van Gog T, Paas F (2014). An embedded and embodied cognition review of instructional manipulatives. Educational Psychology Review.

[CR29] Pouw WTJL, De Nooijer JA, Van Gog T, Zwaan RA, Paas F (2014). Toward a more embedded/extended perspective on the cognitive function of gestures. Frontiers in Psychology.

[CR30] Pouw WTJL, Mavilidi M, Van Gog T, Paas F (2016). Gesturing during mental problem solving reduces eye movements, especially for individuals with lower visual working-memory capacity. Cognitive Processing.

[CR31] Pouw W, Van Gog T, Zwaan RA, Agostinho S, Paas F (2018). Co-thought gestures in children's mental problem solving: Prevalence and effects on subsequent performance. Applied Cognitive Psychology.

[CR32] Shephard S, Metzler D (1988). Mental rotation: effects of dimensionality of object and type of task. Journal of Experimental Psychology: Human Perception and Performance.

[CR33] Smithson L, Nicoladis E (2014). Lending a hand to imagery? The impact of visuospatial working memory interference upon iconic gesture production in a narrative task. Journal of Nonverbal Behavior.

[CR34] So WC, Ching THW, Lim PE, Cheng X, Ip KY (2014). Producing gestures facilitates route learning. PloS one.

[CR35] Stephen DG, Dixon JA, Isenhower RW (2009). Dynamics of representational change: entropy, action, and cognition. Journal of Experimental Psychology: Human Perception and Performance.

[CR36] Trofatter C, Kontra C, Beilock S, Goldin-Meadow S (2014). Gesturing has a larger impact on problem-solving than action, even when action is accompanied by words. Language, Cognition and Neuroscience.

[CR37] Wagner SM, Nusbaum H, Goldin-Meadow S (2004). Probing the mental representation of gesture: Is handwaving spatial?. Journal of Memory and Language.

[CR38] Zook NA, Davalos DB, Delosh EL, Davis HP (2004). Working memory, inhibition, and fluid intelligence as predictors of performance on Tower of Hanoi and London tasks. Brain and Cognition.

[CR39] Zurina H, Williams J (2011). Gesturing for oneself. Educational Studies in Mathematics Education.

